# Binaural Beats Stimulation for Cognitive Enhancement in Alzheimer’s and Related Diseases: An Umbrella Review

**DOI:** 10.1093/geroni/igaf122.2847

**Published:** 2025-12-31

**Authors:** Yawen Li, Zhaojing Chen, Jie Yu, Kevin Zhang

**Affiliations:** California State University, San Bernardino, San Bernardino, California, United States; California State University, San Bernardino, San Bernardino, California, United States; California State University, San Bernardino, San Bernardino, California, United States; California State University, San Bernardino, San Bernardino, California, United States

## Abstract

Binaural beats stimulation (BBS) is a promising neuromodulation technique that utilizes auditory stimulation to influence brainwave activity. This umbrella review synthesizes evidence from multiple systematic reviews evaluating the efficacy of BBS for cognitive enhancement in Alzheimer’s disease (AD) and related neurodegenerative conditions. The methodology adhered to standard umbrella review protocols, involving systematic searches across multiple databases, including Web of Science (WOS), CINAHL, Scopus, PubMed, and PsycINFO. A total of 154 review papers were initially identified, and seven systematic reviews were included following extensive screening and critical appraisal for methodological quality using standardized tools such as AMSTAR 2 or ROBIS. Findings suggest that BBS at 40 Hz enhances cognitive function, improves memory recall, and modulates neural network connectivity. BBS also demonstrates potential in reducing symptoms of anxiety and depression, promoting relaxation, and improving attention and executive function. Physiological benefits include improved sleep quality, increased slow-wave sleep, and potential reduction in amyloid and tau pathology. Despite these promising outcomes, study heterogeneity, lack of standardized protocols, and limited large-scale clinical trials pose challenges in establishing BBS as a clinical intervention. Future research should focus on refining stimulation parameters, conducting robust randomized clinical trials, and integrating BBS with other therapeutic approaches. This review highlights the potential of BBS as a noninvasive intervention for cognitive enhancement and neuroprotection while emphasizing the need for further investigation.

